# Elevated D-dimer Levels in the Exacerbation of End-Stage Chronic Obstructive Pulmonary Disease (COPD) With Hypercapnia

**DOI:** 10.7759/cureus.79574

**Published:** 2025-02-24

**Authors:** Brittany E Reid, Stephen DiGiuseppe, Hesam Akbarian-Tefaghi

**Affiliations:** 1 Osteopathic Medicine, Edward Via College of Osteopathic Medicine, Monroe, USA; 2 Microbiology, Edward Via College of Osteopathic Medicine, Monroe, USA; 3 Hospital Medicine, Willis-Knighton Health System, Shreveport, USA

**Keywords:** chronic obstructive pulmonary disease (copd), d-dimer levels, elevated d-dimer, hypercapnia, pulmonary disease

## Abstract

D-dimer levels can be elevated in a variety of conditions including pulmonary embolism, deep vein thrombosis, disseminated intravascular coagulation, pregnancy, cigarette smoking, and infection. It has been proposed that D-dimer levels in acute exacerbation of chronic obstructive pulmonary disease (AECOPD) can predict short-term and long-term survival. Here, we present a case of an elderly male who presented to the hospital with shortness of breath with an oxygen saturation of 77% on his two liters of home nasal cannula. He had a history of chronic obstructive pulmonary disease (COPD), heart failure with preserved ejection fraction (HFpEF), coronary artery disease, and current tobacco use. Labs indicated an elevated D-dimer level of 5.58 μg/mL. He was admitted to the intensive care unit (ICU) due to severe symptoms, which improved on bilevel positive airway pressure (BiPAP). His chest X-ray showed bilateral interstitial opacities with hyperexpansion. A computed tomography (CT) angiogram of the lungs did not show pulmonary embolism, but chronic bronchitis and severe emphysematous changes were evident. The patient’s elevated D-dimer levels coincided with acute hypercapnic respiratory failure from end-stage (Global Initiative for Chronic Obstructive Lung Disease (GOLD) stage IV) COPD. The patient’s hospital course was complicated by pneumothorax on day 10 of admission. He was managed conservatively and transferred to long-term acute care due to the continued need for BiPAP support and a high-flow nasal cannula. This case and others continue to support that even very high levels of D-dimer in symptomatic patients may not indicate the presence of pulmonary embolism.

## Introduction

The Global Initiative for Chronic Obstructive Lung Disease (GOLD) report states that “chronic obstructive pulmonary disease (COPD) is a heterogeneous lung condition characterized by chronic respiratory symptoms (dyspnea, cough, sputum production and/or exacerbations) due to abnormalities of the airways (bronchitis, bronchiolitis) and/or alveoli (emphysema) that cause persistent, often progressive, airflow obstruction” [1]. This condition was estimated to impact approximately 480 million adults worldwide [2]. They also predict that by 2050, this will increase to 592 million. The World Health Organization (WHO) data from 2021 suggests that COPD is the fourth leading cause of death worldwide [3]. The WHO adds that 70% of COPD in high-income countries is from tobacco use, as opposed to 30-40% in low- to middle-income countries whose populations get COPD from indoor air pollution due to using wood, coal, animal waste, and crop leftovers for heating and cooking.

Due to the prevalence of COPD, it has been estimated to have a significant impact on health economics in the United States [4-9]. One study estimates that COPD had an economic impact (adjusted for comorbidities) of $32.1 billion in 2010 and they predicted by 2020 that it would increase to $49.0 billion in the United States [5]. Another study estimates that healthcare spending on COPD in the United States was mainly due to COPD hospitalization [6]. This study predicts that there will be a 210% increase in COPD hospitalizations and an 182% increase in the duration of inpatient stays between 2010 and 2030. Other studies also show that hospital visits due to acute exacerbation of chronic obstructive pulmonary disease (AECOPD) are the main source of the economic burden [7,8] and AECOPD-related hospital visits continue to rise [9]. When COPD patients experience a sudden increase in symptoms, it is termed AECOPD [10]. These episodes can be caused by various factors such as infection, environment, deep vein thrombosis (DVT), pulmonary embolism (PE), and heart failure. Hypercapnia (elevated levels of retained carbon dioxide), increased age, the need for mechanical ventilation, low body mass index, and prolonged steroid usage were associated with increased mortality for these exacerbations. AECOPD episodes also can increase the risk of myocardial infarctions, stroke, DVT, PE, and infections.

COPD can be diagnosed by pulmonary function tests (PFTs) and computed tomography (CT) scans, which can also be used to monitor disease progression [11]. However, in AECOPD, there have been efforts to study other prognostic measures. There is a significant correlation between AECOPD and elevation of the D-dimer level [1,12-14]. D-dimer is produced when a blood clot is broken down and two fibrin fragments form a dimer [15]. In a fast noninvasive manner with a blood test, D-dimer not only can indicate the presence of venous thromboembolism (including PE and deep vein thrombosis) in emergency settings, but it can also be used for the monitoring of the presence of infection, coagulopathy, and other complications affecting patients in critical care settings [16]. Venous thromboembolism (VTE) is a serious condition and delay in its diagnosis can cause significant morbidity and mortality. Prior studies have shown that patients with COPD are more prone to developing VTE and may have higher baseline D-dimer levels [12-14]. D-dimer can be elevated in COPD due to the role inflammation can play in creating a hypercoagulable state [17]. Our case provides further evidence that D-dimer levels can be even higher in AECOPD. CT pulmonary angiography (CTPA) is the gold standard to differentiate between AECOPD and PTE. However, CTPAs are costly and can cause kidney injury due to contrast use [12]. As the GOLD report also mentioned, there has been evidence to show that preventive therapy with low molecular heparin (after hematological screening) can help reduce the D-dimer levels in AECOPD [1]. Therefore, evidence correlating D-dimer levels and AECOPD can help update diagnostic guidelines to reduce unneeded costly measures [12-14,18,19]. 

The D-dimer cutoff at the hospital the patient presented to was 0.5 μg/mL. Some studies also utilized this cutoff value [12,14], finding higher levels of D-dimer averages in stable COPD [18,19] patients and even higher levels in AECOPD [12-14]. Due to the current cutoff value, these patients undergo the costly PE workup with CTPAs. Increasing the D-dimer cutoff values for AECOPD could prevent unneeded CTPAs. With the prevalence of COPD and studies demonstrating elevated D-dimer levels in AECOPD patients without PE, we present this case so that it may be used for determining the appropriate D-dimer cut-off values for AECOPD patients.

## Case presentation

A 76-year-old male presented to the emergency department with shortness of breath and oxygen saturation of 77% on his two-liter home nasal cannula. The patient had a medical history of COPD, heart failure with preserved ejection fraction (HFpEF), coronary artery disease, hyperlipidemia, current cigarette use, myocardial infarction, and asthma. He had multiple prior admissions for respiratory distress and was previously intubated. Past surgical history included coronary artery bypass surgery and porcine bioprosthetic mitral valve replacement. Family history revealed a deceased father with cancer and a deceased mother with diabetes. Social history was significant for smoking two packs of cigarettes per day for 40 years. His current medications were aspirin, vitamin D3, fluticasone proprion-salmeterol, gabapentin, ipratropium-albuterol, pantoprazole, and simvastatin. The patient reported nausea with Ibuprofen use, but no other allergies. The review of systems was positive for weight loss, cough, and shortness of breath. Vital signs revealed a temperature of 99.4 °F, heart rate of 95 beats per minute, respiratory rate of 24 breaths per minute, blood pressure of 114/83 millimeters of mercury (mm Hg), and oxygen saturation of 98% on bilevel positive airway pressure (BiPAP). Physical examination revealed a cachectic man with sunken eyes, a body mass index of 16.2 kg/m^2^, sternotomy scar, cough, shortness of breath, and bilateral wheezing. The patient did not show any signs of fluid overload, such as leg swelling.

Coagulation studies were significant for a D-dimer of 5.58 μg/mL (Table 1). Significant laboratory results included white blood cell (WBC) of 13.4 10E3/μL, neutrophil % of 88.1, neutrophil # of 11.8 10E3/μL, troponin I of 0.020 ng/mL, B-type natriuretic peptide (BNP) of 239 pg/mL, creatinine of 0.87 mg/dL, blood urea nitrogen (BUN) of 28 mg/dL, anion gap of 6 mmol/L, and lactic acid of 3.8 mmol/L (Table 1). Arterial blood gas (ABG) was significant for pH of 7.28, pCO_2_ of 70.6 mm Hg, pO_2_ of 179 mm Hg, HCO_3_ of 29 mmol/L, base excess of 4.9 mmol/L (Table 1). Urine analysis showed casts, 4-10 / high power field (HPF) red blood cells, 3+ protein, and occult blood (Table 1). Cultures were performed, which revealed a negative urine culture, three negative urine analyses, and four sets of negative blood cultures. A respiratory viral polymerase chain reaction (PCR) was also negative.

**Table 1:** Laboratory results for the hospitalized patient with chronic obstructive pulmonary disease (COPD)

Day of admission arterial blood gas		
Parameters	Values	Reference ranges
pH	7.28	7.35-7.45
pCO_2_	70.6 mm Hg	35-45 mm Hg
pO_2_	179 mm Hg	80-100 mm Hg
HCO_3_	29 mmol/L	22-26 mm Hg
Base excess	4.9 mmol/L	-2 to +2 mmol/L
Day of admission lab results		
Parameters	Values	Reference ranges
White blood cells	13.4 x 10E3/μL	4.5-11.0 x 10E3/μL
Neutrophil %	88.10%	55-70%
Neutrophil #	11.8 x 10E3/μL	2.5-7.0 x 10E3/μL
Troponin I	0.020 ng/mL	0-0.04 ng/mL
Brain natriuretic peptide	239 pg/mL	<100 pg/mL
Creatinine	0.87 mg/dL	0.6-1.2 mg/dL
BUN	28 mg/dL	5-20 mg/dL
Anion gap	6 mmol/L	4-12 mmol/L
Lactic acid	3.8 mmol/L	<2 mmol/L
Day of admission urine analysis		
Parameters	Values	Reference ranges
Casts	Present	None to few
Red blood cells	4-10 / HPF	<3 cells / HPF
Protein	3+	<150 mg/dL
Occult blood	3+	<3 cells / HPF
Day 10 venous blood gas		
Parameters	Values	Reference ranges
pH	7.46	7.35-7.45
pCO_2_	59 mm Hg	35-45 mm Hg
pO_2_	37 mm Hg	82-97 mm Hg
D-dimer levels		
Parameters	Values	Reference ranges
D-dimer (on admission)	5.58 μg/mL	<0.5 μg/mL
D-dimer (four weeks after admission)	1.82 μg/mL	<0.5 μg/mL

**Figure 1:**
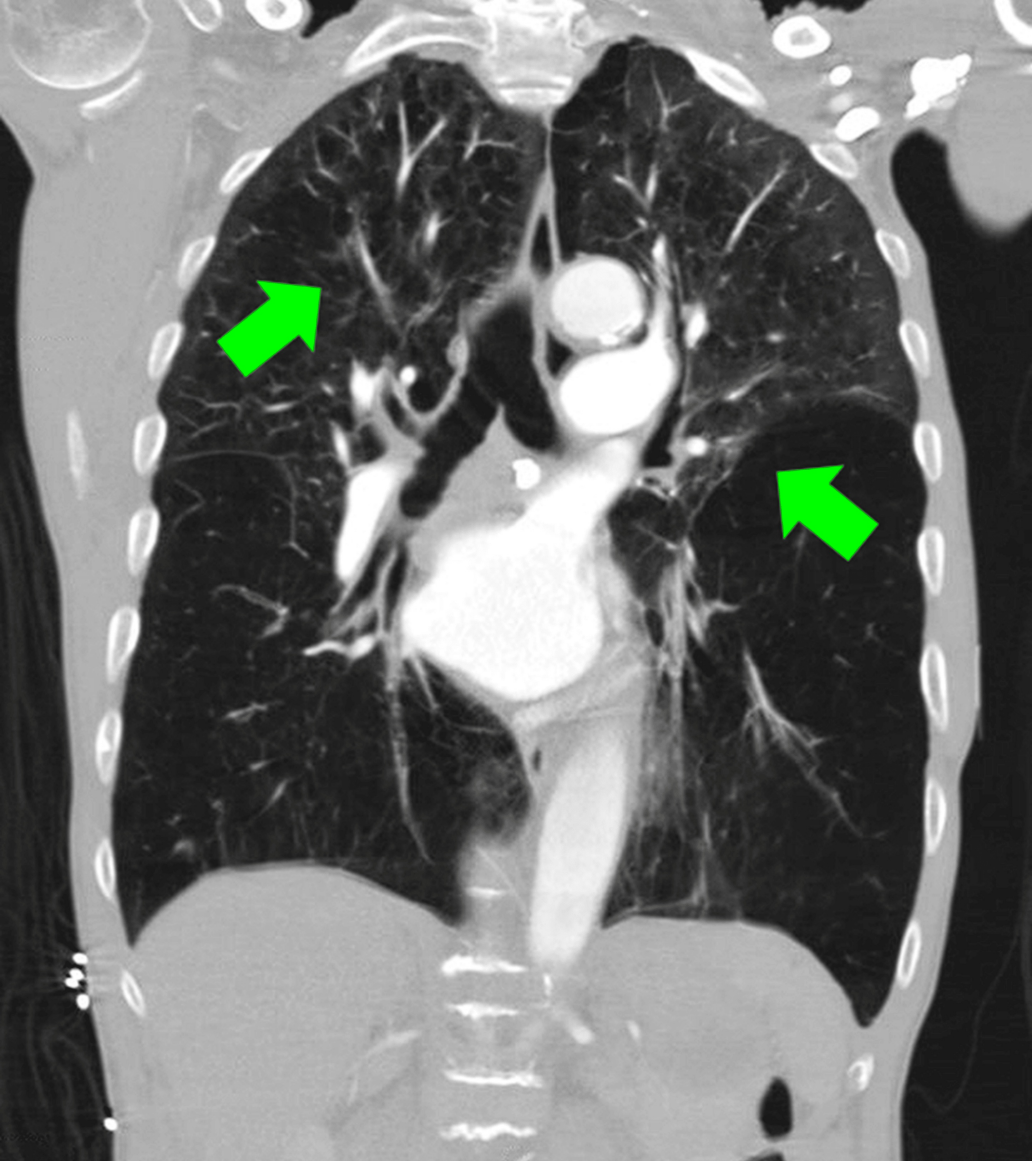
Coronal chest CT angiography (CTA) on admission showing chronic severe emphysema and bronchitis (green arrows).

**Figure 2:**
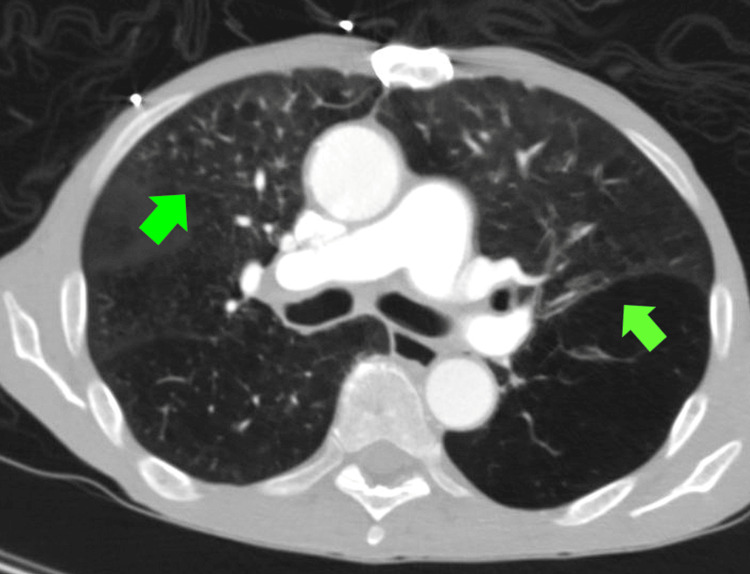
Transverse chest CT angiography (CTA) showing chronic severe emphysema and bronchitis (green arrows).

**Figure 3:**
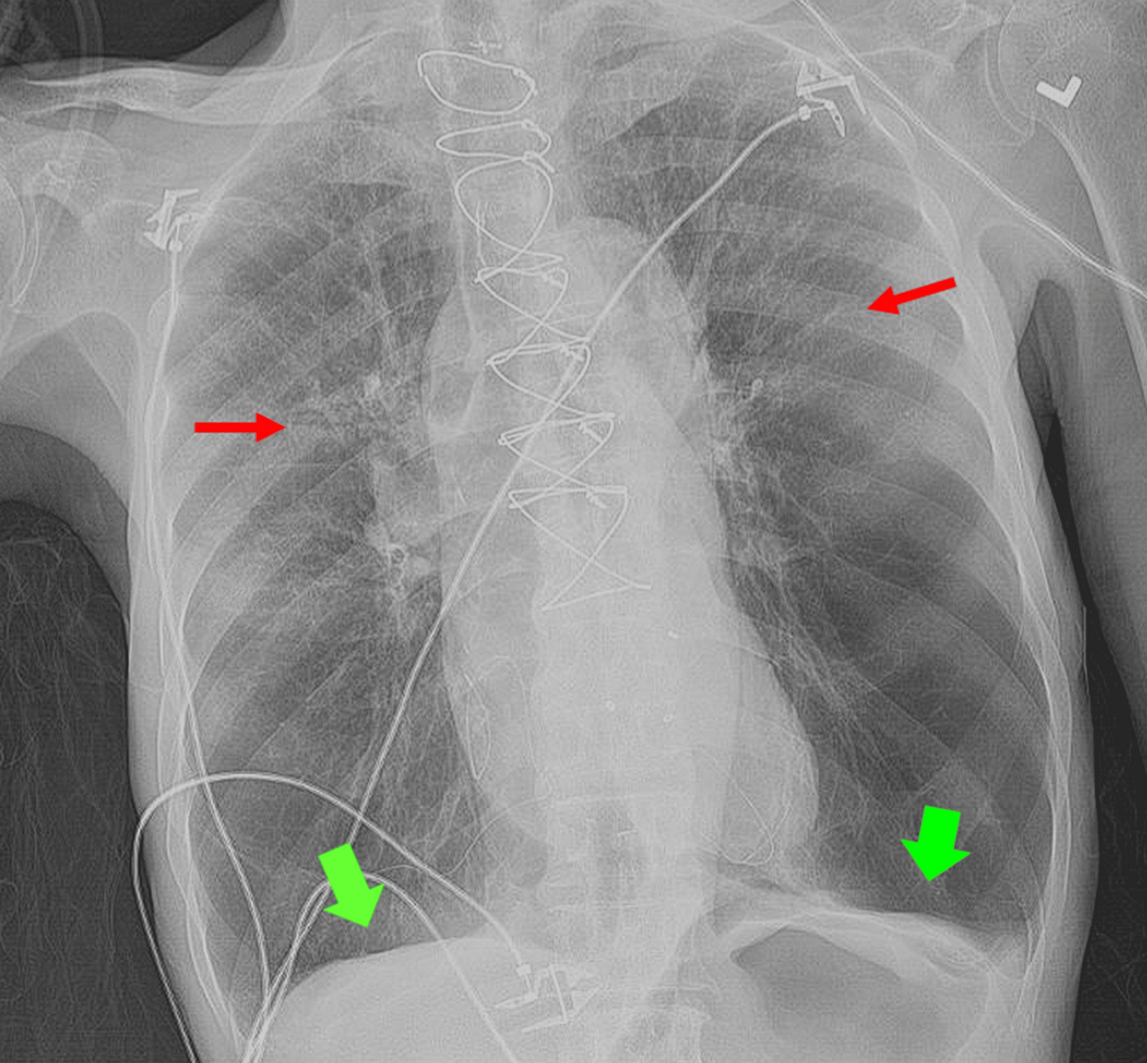
Chest X-ray of the patient on admission. Chest X-ray shows bilateral interstitial opacities (red arrows) and hyperexpansion (green arrows).

**Figure 4:**
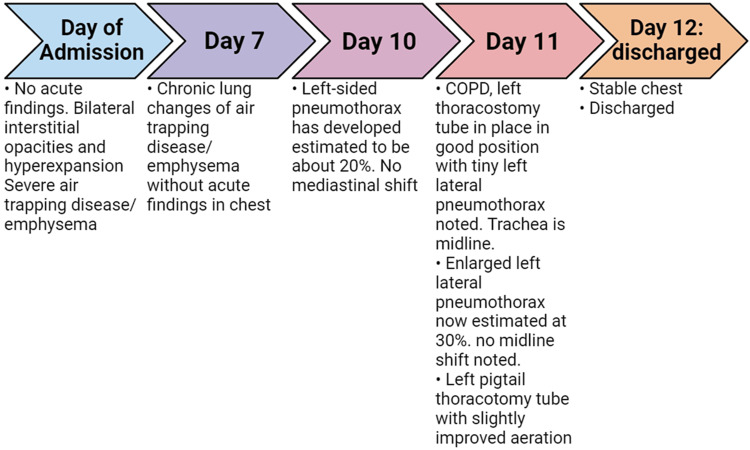
Comprehensive timeline of the X-ray findings from admission to discharge of this patient.

**Figure 5:**
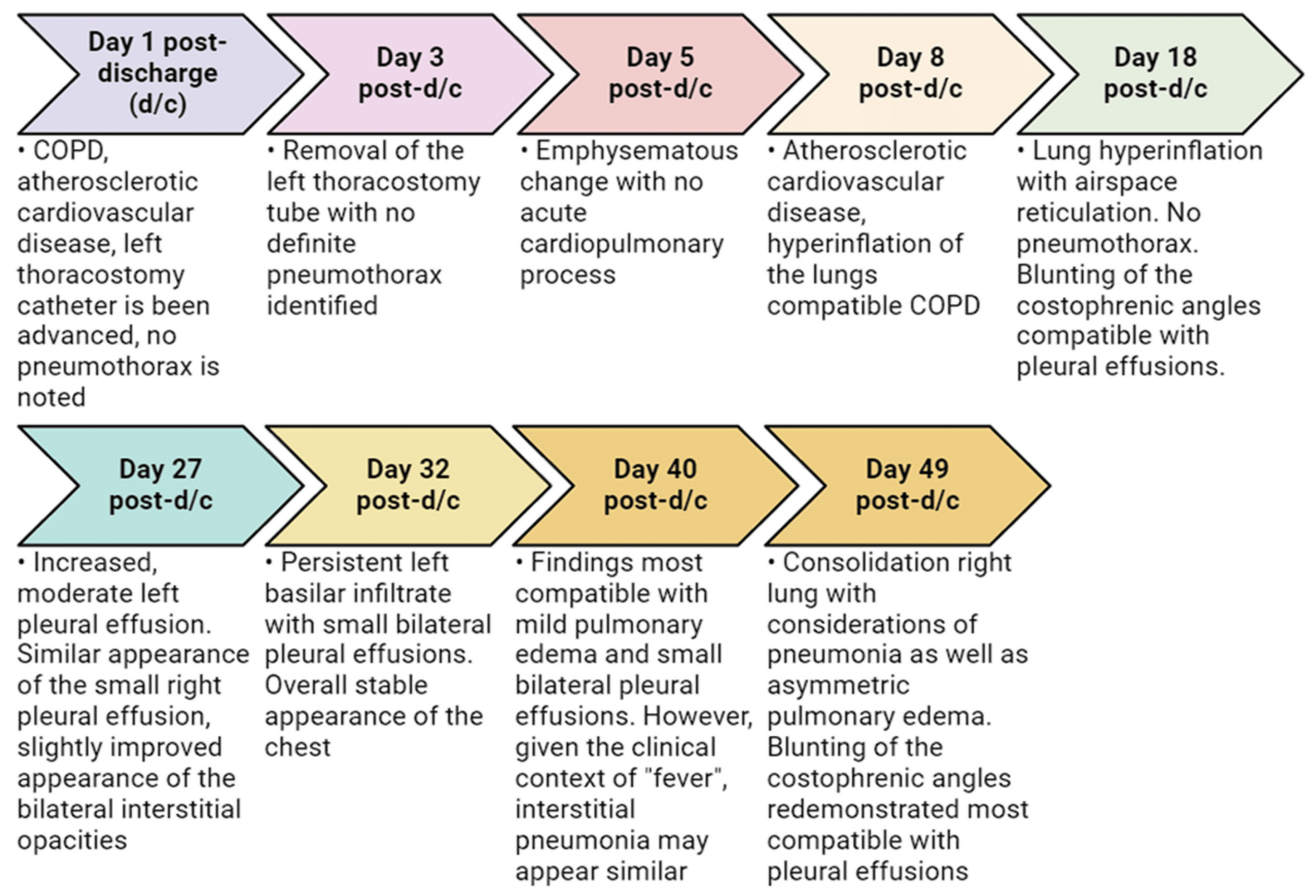
Comprehensive timeline of the X-ray findings since post-discharge day 1 to day 49 for this patient.

Electrocardiogram (EKG) showed no changes from previous admissions. The chest X-ray taken on admission showed bilateral interstitial opacities and hyperexpansion (Figure 1). Computed tomography angiography (CTA) showed findings of chronic severe emphysema and bronchitis and were negative for pulmonary embolism (Figures 2, 3). He was admitted to the hospital with "acute on chronic" hypoxic/hypercapneic respiratory failure secondary to severe COPD exacerbation. This was determined through the physical exam findings aligning with AECOPD (the sudden increase in symptoms and requiring more oxygen support), lab values showing hypercapnia, and imaging that showed chronic COPD. BiPAP was used to help with hypercapnia. Arterial blood gas was trended to monitor improvement. Three days after admission, the patient improved and was transitioned from BiPAP to a high-flow nasal cannula at 30L/min with nighttime BiPAP. The patient became more hypoxic on day 10 and had an increased work of breathing despite BiPAP. The venous blood gas from that evening showed a pH of 7.46, PCO_2_ 59 mm Hg, and PO_2_ 37 mm Hg (Table 1). Chest X-ray was immediately ordered and showed a 20% left-sided pneumothorax with no mediastinal shift. The timeline of the various chest X-rays that were taken over the course of his hospital stay is shown as well (Figures 4, 5). A pigtail chest tube was placed. Goals of care discussions were held with the patient and his family; they wished for a do not intubate/resuscitate (DNI/DNR) status. 

The chest tube was removed five days later without complications. The patient was sent to long-term acute care (LTAC) due to the continued need for BiPAP and >10 L O_2_. Discharge medications included ipratropium-albuterol nebulizer treatments, IV Solu-Medrol, and seven more days of ceftriaxone and azithromycin. It was also advised to continue home medications but to follow up with continued blood work before resuming aspirin. LTAC records show D-dimer levels were repeated four weeks later and had downtrended to 1.82 μg/mL (Table 1). Multiple attempts to ambulate the patient failed due to respiratory distress. As a result, the patient was in LTAC for an extended period, during most of which he was bed-bound. He unfortunately deteriorated later and asked for only comfort care measures to be provided.

## Discussion

Many studies have found elevated D-dimer levels in AECOPD and have focused on utilizing D-dimer levels to determine effective prognostic measures for COPD exacerbations [12-14]. The D-dimer levels taken upon admission for this patient were significantly more elevated than the level measured four weeks later, which suggests that D-dimer levels can be elevated in COPD patients and more so during acute exacerbations.

Alatlı et al. compared fibrinogen and D-dimer levels between 49 patients who presented to the hospital with COPD and 52 patients with no significant past medical history experiencing dyspnea [12]. They found that for patients with COPD, the cut-off D-dimer level was 0.97 μg/mL (p < 0.05), and 6% of COPD patients had a PE found (p < 0.05). Another study reviewed 4,468 patients with AECOPD from seven medical centers over 1.5 years, determining the best cut-off value with the Youden index (a test that estimates diagnostic effectiveness using sensitivity and specificity) [14]. This study found similar results with a D-dimer cut-off value of 0.96 μg/mL (AUC = 0.689), with 2.01% of patients experiencing a VTE within two months of presenting to the hospital. Our patient’s presentation aligns with these studies showing elevated D-dimer levels during AECOPD with negative PE findings. However, this patient’s D-dimer levels were significantly higher than the cut-off values from each of these studies. This supports the research showing that D-dimer cut-off levels to obtain CTA can be increased in AECOPD since his levels were significantly higher and he did not have VTE and there was no identified source of infection on chest X-ray (CXR), CTA, urine analysis, cultures, respiratory viral PCR, or skin exam.

This case is also consistent with studies showing elevated D-dimer levels in stable COPD patients [18,19]. Husebø et al. examined the three-year Bergen COPD Cohort Study with 49 controls, 413 COPD, and 148 AECOPD patients [18]. They found that D-dimer levels were 0.7635 μg/mL in AECOPD patients and 0.4797 μg/mL in stable COPD patients (p < 0.001). Our patient’s D-dimer level decreased but was still elevated even above this study’s level after acute exacerbation. 

Some studies have also shown higher D-dimer levels correlating to short and long-term patient outcomes [19,20]. One study conducted a cross-sectional review that evaluated 90 COPD patients [19]. They found that D-dimer levels were associated with severe COPD exacerbation. Our case is an example of a severe COPD exacerbation, which required a prolonged stay. Another study followed 61 AECOPD patients without VTE for 62.6 months [20]. The results showed higher D-dimer levels in non-survivors (3.18 ± 0.97 μg/mL) than in survivors (1.45 ± 1.18 μg/mL) with p = 0.0006. In-hospital mortality correlated with D-dimer levels >1.52 μg/mL with a sensitivity of 100% and specificity of 63.6%. Our patient had higher D-dimer levels and passed away within 90 days of admission. This patient did have continued tobacco use, which is a significant cause of COPD [3]. With our patient’s presentation and research showing a link between D-dimer and patient outcomes, D-dimer would be useful for estimating patient prognosis.

Since D-dimer is often utilized to determine the diagnostic need for CTPA imaging [12-16, 18, 19], raising the D-dimer cut-off level in AECOPD patients would help reduce unnecessary imaging. By reducing excess imaging, it can help decrease hospital-associated costs since CTPAs are quite expensive [12]. With COPD on the rise and the large impact in-patient care has on the economic burden [6-9], improving diagnostic protocols to reduce costs would be extremely beneficial. Considering a patient’s history, risk factors, and D-dimer levels in the use of imaging is wise due to the risk of PE.

Many studies call for raising the D-dimer cut-off levels for COPD patients [12-14] and this case can contribute to another AECOPD presentation with corresponding D-dimer levels. Using wise clinical judgment and raising the D-dimer cut-off values could help mitigate the economic burden of disease by preventing unnecessary CTPAs. The patient’s presentation supports evidence for increasing D-dimer cut-off values in AECOPD due to elevated levels without another identified cause such as infection, PE, or DVT.

## Conclusions

With COPD being a leading cause of death in the United States, AECOPD poses an increased risk for negative patient outcomes and increased hospital burdens. D-dimer values can be useful for diagnosing and avoiding unnecessary imaging. Our case may provide further support to increase D-dimer cutoff values in AECOPD patients to avoid unnecessary imaging and can provide insight into patient prognosis.
